# Let-7 underlies metformin-induced inhibition of hepatic glucose production

**DOI:** 10.1073/pnas.2122217119

**Published:** 2022-03-28

**Authors:** Di Xie, Fan Chen, Yuanyuan Zhang, Bei Shi, Jiahui Song, Kiran Chaudhari, Shao-Hua Yang, Gary J. Zhang, Xiaoli Sun, Hugh S. Taylor, Da Li, Yingqun Huang

**Affiliations:** ^a^Department of Obstetrics, Gynecology & Reproductive Sciences, Yale University School of Medicine, New Haven, CT 06510;; ^b^Yale Center for Molecular and Systems Metabolism, Yale University School of Medicine, New Haven, CT 06520;; ^c^Medical Basic Experimental Teaching Center, China Medical University, Shenyang 110004, China;; ^d^Center of Reproductive Medicine, Shengjing Hospital of China Medical University, Shenyang 110004, China;; ^e^Department of Pharmacology and Neuroscience, Institute for Healthy Aging, University of North Texas Health Science Center, Fort Worth, TX 76107

**Keywords:** metformin, Let-7, TET3, liver, diabetes

## Abstract

Metformin, the frontline antidiabetic drug, has gained increasing attention for the prevention and treatment of aging, cancer, and cardiovascular disease. Yet a clear mechanistic understanding of its action is still lacking, largely due to the suprapharmacological concentrations of metformin used in most studies. Here, we report an inhibition of glucose production by primary hepatocytes from dietary and genetic mouse models of type 2 diabetes (T2D) using metformin at clinically relevant concentrations. Mechanistically, metformin up-regulates microRNA let-7 that in turn down-regulates TET3, evoking a change in the ratio of hepatocyte nuclear factor 4 alpha (HNF4α) isoforms and subsequent inhibition of key gluconeogenic genes. Importantly, this let-7–mediated mechanism is faithfully recapitulated in mice with T2D chronically treated with therapeutic doses of metformin. Furthermore, hepatic delivery of let-7 ameliorates hyperglycemia and improves glucose homeostasis in diabetic mice, whereas liver-specific inhibition of let-7 abrogates these beneficial effects of metformin. Moreover, let-7 overexpression decreases glucose production from primary hepatocytes from obese humans. Thus, we propose the reactivation of a let-7–dependent pathway that is pathologically repressed in the liver of diabetes as a major mechanism of metformin action and that liver-specific delivery of let-7 represents a potential therapeutic for T2D. Our findings are also pertinent to the development of therapeutic strategies for other chronic diseases.

## Significance

A clear mechanistic understanding of metformin’s antidiabetic effects is lacking. This is because suprapharmacological concentrations of metformin have been used in most studies. Using mouse models and human primary hepatocytes, we show that metformin, at clinically relevant doses, suppresses hepatic glucose production by activating a conserved regulatory pathway encompassing let-7, TET3, and a fetal isoform of hepatocyte nuclear factor 4 alpha (HNF4α). We demonstrate that metformin no longer has potent antidiabetic actions in a liver-specific let-7 loss-of-function mouse model and that hepatic delivery of let-7 ameliorates hyperglycemia and improves glucose homeostasis. Our results thus reveal an important role of the hepatic let-7/TET3/HNF4α axis in mediating the therapeutic effects of metformin and suggest that targeting this axis may be a potential therapeutic for diabetes.

Metformin has been available for the treatment of type 2 diabetes (T2D) for over a half century, owing to its robust efficacy in glycemic control and an excellent safety profile. Metformin elicits its primary antidiabetic action via suppression of hepatic glucose production (HGP) (1–3), although other actions including alteration of gut microbiota have also been documented (4). Various mechanisms have been put forward to explain metformin’s action of suppression of HGP. In the past, much work has focused on the inhibition of mitochondrial respiratory chain complex 1 and the subsequent reduction in cellular energy charge and activation of the energy sensor AMPK (1–3). However, these mechanisms have been challenged because complex I inhibition and AMPK activation are often observed in the context of suprapharmacological concentrations of metformin (1, 3). Recently, reduction-oxidation (redox)-dependent mechanisms have been proposed suggesting that metformin modulates cellular redox balance resulting in HGP suppression in a complex I inhibition/AMPK activation-independent manner; yet, how cellular redox regulates HGP has remained the subject of debate (3, 5, 6). In recent years, repurposing metformin for the prevention and/or treatment of other illnesses including cancer, cardiovascular disease, and aging has received increased attention (3, 7, 8). Therefore, a clear mechanistic understanding of metformin’s action is pivotal to the development of therapeutic strategies for these chronic diseases.

The evolutionarily conserved transcription factor hepatocyte nuclear factor 4 alpha (HNF4α) acts in concert with coactivator PGC-1α to transcriptionally activate the rate-limiting enzymes G6PC and PEPCK (encoded by *G6pc* and *Pck1*, respectively) in promoting HGP (9). *HNF4α* has two promoters, namely, P2 and P1, driving multiple HNF4α isoforms via alternative splicing. In the liver, the P2 promoter is active during fetal development but is switched off and replaced by transcription from the P1 promoter after birth (10, 11). We have recently reported reactivation of the P2 promoter with an essential role in the control of HGP in the adult liver (12). The activity of the P2 promoter is acutely induced upon fasting and is chronically elevated in T2D. The promoter reactivation occurs in response to glucagon-stimulated up-regulation of TET3, which is specifically recruited to the P2 promoter via interaction with transcription factor FOXA2 (12). TET3 is a member of a family of DNA demethylases that oxidize 5-methylcytosine to 5-hydroxymethylcytosine (5hmC), leading to DNA demethylation (13, 14). Binding of TET3 to the P2 promoter of *HNF4α* induces 5hmC modification and subsequent demethylation, activating P2-dependent transcription (12). Using hyperinsulinemic/euglycemic clamp and liver-specific small interfering RNA delivery, we demonstrated a mechanistic link between pathological activation of the TET3/HNF4α-P2 axis and abnormally augmented HGP that underlies the chronic hyperglycemia in T2D (12).

In this work, we show that metformin, at clinically relevant concentrations, targets the TET3/HNF4α-P2 axis to inhibit HGP through the induction of let-7 using cell and mouse models of T2D and primary hepatocytes from obese humans. Also, we identify let-7 as a potential therapeutic for T2D.

## Results

### Therapeutic Concentrations of Metformin Inhibit HGP.

Following oral administration, metformin is distributed into multiple organs and excreted unchanged in the urine. A similar biodistribution pattern of metformin has been observed in humans and rodents (15–19). The therapeutic concentrations of metformin in human plasma ranged from 0.1 to 20.7 (median 4.5) μM (20–23). After patients with prediabetes were treated with metformin at 1,500 mg/day for 15 wk, a mean plasma metformin concentration of 5.41 μM was obtained (24). Using a physiologically based pharmacokinetic model of mice, the simulated mean metformin concentrations were reported to be 3.5 μM, 6.5 μM, and 9.2 μM in the plasma and 29.2 μM, 53.6 μM, and 76.8 μM in the liver, following a therapeutic daily dosing regimen of 1,000 mg, 2,000 mg, and 3,000 mg in humans, respectively (19). In rodents, chronic oral administration of metformin in drinking water at 200 to 300 mg/kg per day resulted in steady-state plasma metformin concentrations in the range of 5 to 20 μM and hepatic metformin concentrations of ∼40 μM, which is comparable to metformin concentrations achieved in humans with T2D chronically treated with metformin (5, 7, 15). In light of recent reports that chronic metformin administration in healthy, nondiabetic individuals does not alter whole-body glucose homeostasis (25, 26), we acknowledge the importance of studying metformin mechanisms in the context of cell and animal models with compromised (as opposed to normal) intracellular metabolite homeostasis.

**Fig. 1.**
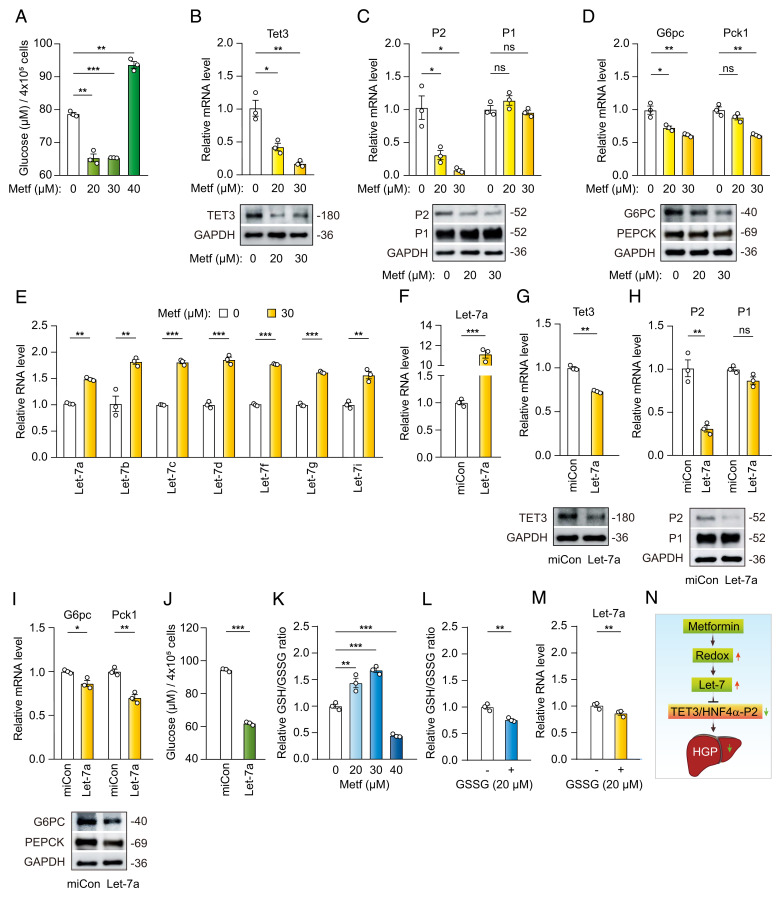
Metformin targets let-7/TET3/HNF4α-P2 in vitro. (*A*) Glucose production by HFD hepatocytes at 40 h following incubation with metformin at the indicated concentrations. (*B*) qPCR (*Top*) and Western blotting (*Bottom*) of TET3 from HFD hepatocytes treated with metformin for 38 h. Protein sizes in kDa are marked on the *Right*. (*C*) qPCR (*Top*) and Western blotting (*Bottom*) of HNF4α-P2 and HNF4α-P1 from HFD hepatocytes treated with metformin for 38 h. (*D*) qPCR (*Top*) and Western blotting (*Bottom*) of G6PC and PEPCK from HFD hepatocytes treated with metformin for 38 h. (*E*) qPCR of let-7 family members from HFD hepatocytes treated with metformin at 30 μM for 24 h. (*F*) qPCR of let-7a from HFD hepatocytes transfected with miCon (nontargeting miRNA) or let-7a for 24 h. (*G*) qPCR (*Top*) and Western blotting (*Bottom*) of TET3 from HFD hepatocytes transfected with miCon or let-7a for 38 h. (*H*) qPCR (*Top*) and Western blotting (*Bottom*) of HNF4α-P2 and HNF4α-P1 from HFD hepatocytes transfected with miCon or let-7a for 38 h. (*I*) qPCR (*Top*) and Western blotting (*Bottom*) of G6PC and PEPCK from HFD hepatocytes transfected with miCon or let-7a for 38 h. (*J*) Glucose production by HFD hepatocytes at 40 h following transfection with miCon or let-7a. (*K*) Relative GSH/GSSG ratio in HFD hepatocytes treated with indicated concentrations of metformin for 18 h. The GSH/GSSG ratio in vehicle-treated cells is arbitrarily set as 1. (*L*) Relative GSH/GSSG ratio in HFD hepatocytes treated with (+) or without (−) 20 μM of GSSG for 5 h. (*M*) qPCR of let-7a from HFD hepatocytes treated with (+) or without (−) 20 μM of GSSG for 24 h. (*N*) A proposed pathway. All data are representative of at least three independent experiments. Error bars are mean with SEM of technical replicates (*n* = 3). *P < 0.05; **P < 0.01; ***P < 0.001; ns, nonstatistical significance. (*A*–*D* and *K*), One-way ANOVA with Dunnett posttest; (*E*–*J*, *L*, and *M*), two-tailed Student’s *t* tests.

To study the therapeutic effects of metformin in vitro, we isolated primary hepatocytes from high fat diet (HFD)-induced diabetic mice (*SI Appendix*, Fig. S1) fed ad libitum and allowed them to adhere for ∼4 h on plates before adding metformin. Metformin treatment was carried out in a complete hepatocyte culture medium containing glucose, insulin, and fetal bovine serum [conditions optimized for cell viability (12)], followed by glucose production assays. As seen in Fig. 1*A*, metformin elicited a nonlinear effect; it reduced glucose production by ∼15% at 20 μM and 30 μM but increased it by ∼15% at 40 μM. Given that metformin treatment led to a 15 to 30% reduction in HGP in patients with T2D (27–29), we considered 30 μM being the cutoff therapeutic concentration for further mechanistic studies in primary hepatocytes isolated from HFD mice.

### Metformin Targets the TET3/HNF4α-P2 Axis.

We have previously documented abnormally increased hepatic expression of TET3 in both humans and mice with T2D (12). Increased TET3 protein epigenetically stimulates the production of HNF4α-P2 (but not HNF4α-P1) leading to elevated expression of G6PC and PEPCK and subsequent augmentation of HGP (12). The mechanistic connection between pathological activation of the TET3/HNF4α-P2 axis and heightened HGP was further confirmed using hyperinsulinemic/euglycemic clamp studies (12). To test whether metformin affects the TET3/HNF4α-P2 axis, HFD hepatocytes were incubated with metformin followed by gene expression analysis. Metformin decreased the expression of TET3 (Fig. 1*B*), HNF4α-P2 (without affecting HNF4α-P1) (Fig. 1*C*), G6PC, and PEPCK (Fig. 1*D*). Taken together with glucose output data (Fig. 1*A*), these results suggest that metformin inhibits gluconeogenic gene expression and HGP at least in part by repressing the TET3/HNF4α-P2 pathway.

### Metformin Up-Regulates let-7.

To begin delineating the regulatory signals induced by metformin and that are upstream of TET3/HNF4α-P2, we first looked into let-7. Both human and mouse TET3 mRNAs contain multiple binding sites for let-7 in their coding regions and are validated targets for let-7-mediated posttranscriptional inhibition of expression (30). Thus, HFD hepatocytes were treated with metformin, and let-7 expression levels were assessed. Metformin increased the expression of multiple let-7 family members reported to be expressed in the murine liver (31) (Fig. 1*E*). These results demonstrate that metformin increases the expression of let-7.

### Let-7 Inhibits HGP.

Next, we tested whether let-7 overexpression was sufficient to suppress TET3/HNF4α-P2 and HGP. mRNA target recognition by microRNAs is largely dictated by “seed” sequences (nucleotides 2 to 7 in mature microRNAs) (32). All mature let-7 isoforms share the same seed sequence. Thus, we transfected a single let-7 isoform, let-7a, into HFD hepatocytes to achieve an ∼11-fold overexpression of let-7a (Fig. 1*F*), mimicking the sum of all let-7 isoforms induced by metformin (Fig. 1*E*). Let-7a overexpression decreased the expression of TET3 (Fig. 1*G*), HNF4α-P2 (but not HNF4α-P1; Fig. 1*H*), G6PC, and PEPCK (Fig. 1*I*) and decreased glucose production (Fig. 1*J*). Given that metformin up-regulates let-7 (Fig. 1*E*), these results point to let-7 as a potentially important mediator of metformin in the regulation of TET3/HNF4α-P2 and HGP.

### Hepatic Redox Affects let-7 Expression.

The redox state in a cell is determined by a fine-tuned balance between oxidant production and the antagonistic action of antioxidant machinery. Reactive oxygen species (ROS) are the most prevalent oxidants that are byproducts of aerobic metabolism. The antioxidant machinery is composed of enzymatic and nonenzymatic antioxidants. Among the nonenzymatic antioxidants, the widely distributed redox pair glutathione (GSH) and its oxidized form (GSSG) serve as the main redox guardians, and the ratio of GSH/GSSG has been commonly used as a readout for cell and tissue redox status (33–35). microRNA biogenesis begins with the transcription of primary microRNAs, followed by processing in the nucleus by Drosha to give precursor miRNAs (premiRNAs). After being transported to the cytoplasm, premiRNAs are further cleaved by Dicer to generate mature microRNAs (36). It has been well established that the cellular redox status modulates microRNA expression (37–39). For example, by regulating the expression and activity of components of the microRNA biogenesis machinery including DGCR8, Drosha, exportin 5, and Dicer, ROS affects the expression levels of mature microRNAs (39).

Metformin has been suggested to increase the cytosolic redox potential in hepatocytes (5, 6, 40). We hypothesized that metformin modulates hepatic redox, thereby affecting let-7 expression. To test this hypothesis, HFD hepatocytes were incubated with metformin, followed by redox assessment using the GSH/GSSG ratio. As shown in Fig. 1*K*, metformin increased the GSH/GSSG ratio at concentrations of 20 μM and 30 μM and decreased it at 40 μM. Notably, there was a nonlinear relationship between the redox state of the hepatocytes and the maximal dose of metformin inhibiting glucose production (compare between Fig. 1 *A* and *K*). Furthermore, treating HFD hepatocytes with GSSG in the absence of metformin decreased the GSH/GSSG ratio (Fig. 1*L*), with a concomitant decrease in let-7 expression (Fig. 1*M*). These results are in line with the notion that metformin up-regulates let-7 likely in part by enhancing the hepatic redox state.

### Metformin Targets let-7/TET3/HNF4α-P2 in ob/ob Hepatocytes.

Using HFD hepatocytes, we were able to demonstrate that metformin decreases HGP at least in part by suppressing the TET3/HNF4α-P2 axis and that it does so by increasing let-7 expression (Fig. 1*N*). Next, we performed experiments in hepatocytes isolated from a second mouse model of T2D, the leptin-deficient ob/ob mice, and showed that this regulatory pathway (Fig. 1*N*) also appears active in these cells (*SI Appendix*, Figs. S2 and S3).

### Metformin Targets let-7/TET3/HNF4α-P2 In Vivo.

**Fig. 2.**
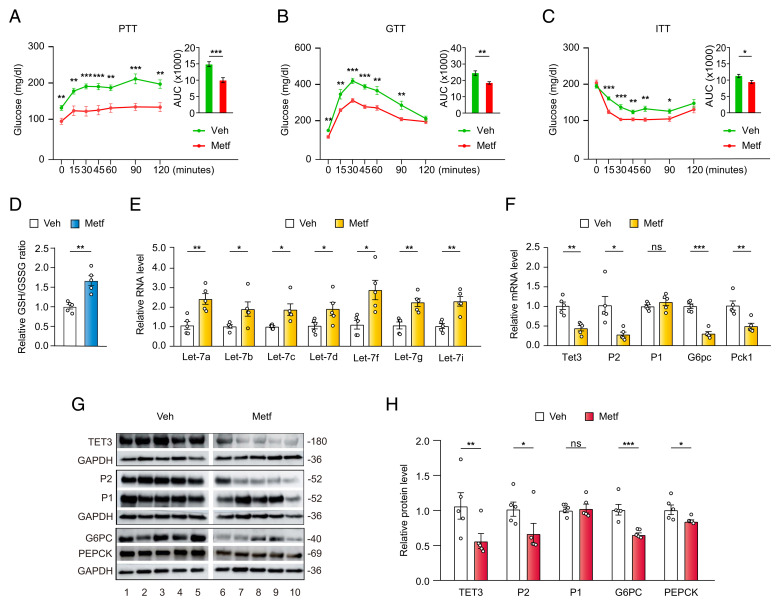
Metformin targets let-7/TET3/HNF4α-P2 in vivo. (*A*) PTT in HFD mice treated with vehicle or metformin for 3 wk. (*B*) GTT in HFD mice treated with vehicle or metformin for 4 wk. (*C*) ITT in HFD mice treated with vehicle or metformin for 5 wk. (*D*) Relative GSH/GSSG ratio in liver tissues from HFD mice treated with vehicle or metformin for 6 wk. (*E*) qPCR of let-7 isoforms in liver tissues from HFD mice treated with vehicle or metformin for 6 wk. (*F*) qPCR of indicated genes in liver tissues from HFD mice treated with vehicle or metformin for 6 wk. (*G*) Western blotting of liver tissues from HFD mice treated with vehicle or metformin for 6 wk. Each lane represents one individual mouse. (*H*) Quantification of *G*, *n* = 5, and each dot represents one mouse. All data are presented as mean of SEM. *P < 0.05; **P < 0.01; ***P < 0.001; ns, not statistically significant. (*A*–*C*) *n* = 8 animals in each group, two-way ANOVA with Sidak posttest; (*D*–*F* and *H*) *n* = 5 animals in each group, two-tailed Student’s *t* tests.

To address whether the let-7/TET3/HNF4α-P2 regulatory pathway operates in vivo, metformin was orally administrated to HFD mice in drinking water at a daily dose of 200 mg/kg, followed by metabolic evaluation 3 wk later. This dosing regimen resulted in mean steady concentrations of 8.63 μM in the plasma and 35.69 μM in the liver (*SI Appendix*, Table S1), which are comparable to the therapeutic concentrations found in humans (5, 19–23). Metformin treatment expectedly decreased HGP, as determined by pyruvate tolerance tests (PTTs) (Fig. 2*A*). This was accompanied by improved systemic glucose metabolism as assessed by glucose and insulin tolerance tests (GTTs and ITTs) (Fig. 2 *B* and *C*). Notably, metformin treatment shifted liver tissue redox to a more reduced state (Fig. 2*D*). When hepatic gene expression was analyzed, increased expression of let-7 (Fig. 2*E*) in metformin- vs. vehicle-treated animals was observed, consistent with metformin-induced up-regulation of let-7. Furthermore, there was a decrease in the expression of TET3, HNF4α-P2 (but not HNF4α-P1), G6PC, and PEPCK (Fig. 2*F*), consistent with repression of the TET3/HNF4α-P2 pathway. This metformin-induced alteration of gene expression was also reflected at the protein level (Fig. 2 *G* and *H*). These results suggest that chronic metformin therapy up-regulates let-7 leading to the suppression of the TET3/HNF4α-P2 axis and HGP in vivo.

### Let-7 Loss-of-Function Abrogates Metformin-Induced HGP Repression In Vivo.

**Fig. 3.**
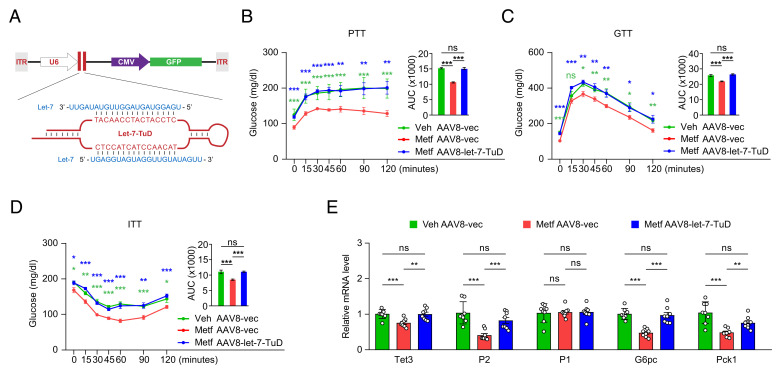
Inhibition of let-7 abolishes metformin-induced HGP repression in vivo. (*A*) Schematic of Let-7-TuD-expressing AAV construct. ITR, inverted terminal repeat; U6, U6 promoter driving the expression of Let-7-TuD. The cytomegalovirus (CMV) promoter drives the expression the GFP reporter. The Let-7-TuD forms an imperfect RNA hairpin containing two opposing sequences (red capital letters) that bind specifically to two molecules of let-7 (blue capital letters). (*B*) PTT in HFD mice treated with vehicle plus AAV8-vec, metformin plus AAV8-vec, or metformin plus AAV8-let-7-TuD for 2 wk. (*C*) GTT at 3 wk after the indicated treatments. (*D*) ITT at 4 wk after the indicated treatments. (*E*) qPCR of indicated genes in liver tissues from mice at 4.5 wk after the indicated treatments. All data are presented as mean of SEM (*n* = 8 animals in each group). *P < 0.05; **P < 0.01; ***P < 0.001; ns, not statistically significant. Two-way ANOVA with Sidak posttest.

To address whether let-7 is a major mediator of metformin in HGP regulation, we treated diabetic mice with metformin in the presence of a let-7 Tough Decoy (Let-7-TuD) predicted to inhibit the activity of all let-7 family members (Fig. 3*A*). TuDs are RNA molecules transcribed from RNA polymerase III that fold into an imperfect RNA hairpin that harbors two opposing microRNA binding sites in a bulge (Fig. 3*A*); TuDs bind target microRNAs thereby inhibiting their function (41). TuDs expressed from viral vectors can induce potent and long-lasting suppression of microRNA activity in mammalian cells (41–44). Thus, HFD mice were infused via tail vein with Let-7-TuD-expressing adeno-associated virus vector serotype 8 (AAV8-let-7-TuD) (Fig. 3*A*) or control AAV (AAV8-vec), in addition to metformin therapy using the same regimen (daily dose of 200 mg/kg in drinking water). We and others have previously documented high levels of liver-specific transgene expression in murine models using AAV8-mediated systemic delivery (12, 45–47). If let-7 activity was required for metformin-induced HGP suppression and improvement of glucose homeostasis, we would predict a loss of these beneficial effects of metformin in mice treated with AAV8-let-7-TuD as compared to those treated with AAV8-vec. Results (Fig. 3 *B*–*D*) support this prediction. A gene expression analysis further confirmed derepression of the TET3/HNF4α-P2 axis by AAV8-let-7-TuD (Fig. 3*E*). Importantly, the loss of the beneficial effects of metformin as a result of let-7 inhibition was specific to metformin, as liver toxicity, as measured by plasma levels of alanine transaminase (ALT), aspartate transaminase (AST), lactate dehydrogenase (LDH), and bilirubin, was not observed (*SI Appendix*, Table S2). Based on these results, we conclude that let-7 is a key downstream effector of metformin in the regulation of HGP.

### Hepatic Delivery of let-7 Improves Glucose Homeostasis in Diabetic Mice.

**Fig. 4.**
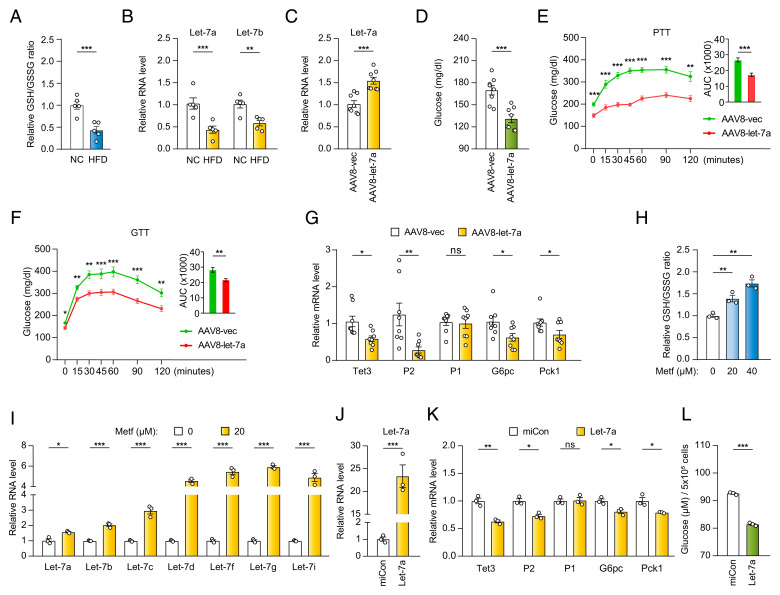
Hepatic let-7 overexpression improves glucose homeostasis. (*A*) Relative GSH/GSSG ratio in liver tissues from mice fed with NC or HFD for 12 wk. *n* = 5 animals per group, two-tailed Student’s *t* tests. (*B*) qPCR of let-7a and let-7b in liver tissues from mice fed with NC or HFD for 12 wk. *n* = 5 animals per group, two-tailed Student’s *t* tests. (*C*) qPCR of let-7a in liver tissues from ob/ob mice 6 wk after injection with AAV8-let-7a or AAV8-vec. *n* = 8 animals in each group, Two-tailed Student’s *t* tests. (*D*) Fasting glucose in ob/ob mice 5 wk after injection with AAV8-let-7a or AAV8-vec. *n* = 8 animals in each group, two-tailed Student’s *t* tests. (*E*) PTT in ob/ob mice 3 wk after injection with AAV8-let-7a or AAV8-vec. *n* = 8 animals in each group, two-way ANOVA with Sidak posttest. (*F*) GTT in ob/ob mice 4 wk after injection with AAV8-let-7a or AAV8-vec. *n* = 8 animals in each group, two-way ANOVA with Sidak posttest. (*G*) qPCR of indicated genes in liver tissues from ob/ob mice 6 wk after injection with AAV8-let-7a or AAV8-vec. *n* = 8 animals in each group, two-tailed Student’s *t* tests. (*H*) Relative GSH/GSSG ratio in primary hepatocytes from a female obese human treated with indicated concentrations of metformin for 18 h. *n* = 3, technical replicates, One-way ANOVA with Dunnett posttest. (*I*) qPCR of let-7 in primary hepatocytes from obese humans treated with metformin for 24 h. *n* = 3, technical replicates, two-tailed Student’s *t* tests. (*J*) qPCR of let-7a in primary hepatocytes from obese humans transfected with miCon or let-7a for 24 h. *n* = 3, technical replicates, two-tailed Student’s *t* tests. (*K*) qPCR of indicated genes in primary hepatocytes from obese humans transfected with miCon or let-7a for 36 h. *n* = 3, technical replicates, two-tailed Student’s *t* tests. (*L*) Glucose production by primary hepatocytes from obese humans transfected with miCon or let-7a for 38 h. *n* = 3, technical replicates, two-tailed Student’s *t* tests. All data are presented as mean of SEM. *P < 0.05; **P < 0.01; ***P < 0.001; ns, not statistically significant.

Our model (Fig. 1*N*) predicts a repression of the redox/let-7 axis in diabetic liver. Indeed, there was a decrease in the GSH/GSSG ratio (Fig. 4*A*) as well as in the expression of let-7 (Fig. 4*B*) in the liver of mice fed with HFD as compared to normal chow (NC). Our model also predicts that liver-specific let-7 overexpression would decrease HGP and reverse hyperglycemia and glucose intolerance in diabetic mice. As metformin targets let-7/TET3/HNF4α-P2 in primary hepatocytes both from HFD and ob/ob mice, we tested the hypothesis using ob/ob mice as a proof-of-principle. Thus, ob/ob mice were systemically administrated with let-7a-expressing AAV (AAV8-let-7a) or AAV8-vec, followed by metabolic analyses 3 wk later. Hepatic let-7a overexpression (Fig. 4*C*) decreased fasting glucose (Fig. 4*D*) and blood glucose in PTT (Fig. 4*E*) and GTT (Fig. 4*F*), demonstrating reduced HGP and improved glucose homeostasis. Gene expression analysis revealed a suppression of the TET3/HNF4α-P2 axis in let-7a overexpressed vs. control livers (Fig. 4*G*).

As suggested by the conservation of the TET3/HNF4α-P2 pathway in the regulation of HGP in human and mouse (12), metformin treatment of primary hepatocytes from a female obese human enhanced redox (Fig. 4*H*) and increased let-7 expression (Fig. 4*I*). Moreover, let-7a overexpression (to a level comparable to that of all let-7 family members combined, Fig. 4*J*) led to a repression of the TET3/HNF4α-P2 axis (Fig. 4*K*) and decreased glucose production (Fig. 4*L*). Similar results were obtained from primary hepatocytes from a male obese human (*SI Appendix*, Fig. S4). Collectively, our results reveal a pathological repression of the conserved let-7-dependent pathway in the liver of diabetes that is reactivated by metformin.

## Discussion

The redox-dependent model suggests that metformin inhibits HGP by increasing the hepatocellular redox state. Specifically, Madiraju et al. (5, 40) proposed that metformin directly inhibits mG3PDH, an enzyme involved in the glycerol-phosphate shuttle, leading to an increase in the cytosolic redox potential that impedes gluconeogenesis from glycerol and reduced (but not oxidized) gluconeogenic substrates. Alshawi et al. (6), on the other hand, suggested that metformin-induced inhibition of the malate-aspartate shuttle (through mitochondrial depolarization caused by metformin’s positive charge) produces two outcomes, as follows: first, an increase in the cytosolic redox state, and second, stimulation of the glycerol-phosphate shuttle leading to activation of PFK1 (a key enzyme in glycolysis) and inhibition of gluconeogenesis via partitioning of substrates toward glycolysis. Despite the contradictory conclusions of these studies, with one (5, 40) suggesting inhibition and the other (6) proposing stimulation of the glycerol-phosphate shuttle, both groups nonetheless pointed to a role of metformin in the modulation of hepatic redox. Using two mouse models of T2D, we demonstrate that metformin enhances hepatic redox and inhibits HGP, at least in part, via activating an evolutionarily conserved regulatory pathway encompassing let-7 and TET3/HNF4α-P2 (Fig. 1*N*). Although metformin can have effects on other tissues/organs, the fact that liver-specific let-7 inhibition is sufficient to abrogate the beneficial effects of metformin on glucose homeostasis (Fig. 3) highlights the pivotal role of hepatic let-7/TET3/HNF4α-P2 in mediating the therapeutic effects of metformin. The critical role of let-7 is further substantiated by the notion that let-7 overexpression suppresses glucose production in primary hepatocytes both from mice and humans with T2D and that hepatic-specific let-7 delivery alleviates hyperglycemia and improves glucose metabolism in mice with diabetes.

Our studies have several limitations. First, the culture conditions for primary hepatocytes we used did not perfectly recapture in vivo conditions and contained nutrients and hormones (e.g., glucose, glucocorticoids and insulin) that could affect glucose production. Thus, we must interpret our in vitro data with caution and in the context of in vivo studies. Second, the PTT is an indirect readout for HGP, which would be of little value without substantive additional results that more directly link data to effects on HGP. However, we did use a hyperinsulinemic/euglycemic clamp, a widely accepted gold standard method for assessing insulin action and HGP in vivo, to confirm the mechanistic involvement of the TET3/HNF4α-P2 axis in HGP regulation (12).

Two important studies reported that whole-body let-7 overexpression in mice induced glucose intolerance (48, 49) and that global let-7 knockdown prevented and treated impaired glucose tolerance (49). As muscle- or pancreas-specific let-7 overexpression induced glucose intolerance (48, 49), the potentially beneficial effects of hepatic let-7 overexpression in the whole-body let-7 overexpression mice were likely masked by the detrimental effects of let-7 overexpression from both the muscle and pancreas. Thus, it is quite likely that the discrepancies between these and our findings are due to the confounding effects of global let-7 overexpression/knockdown. Our results underscore the importance of tissue-specific effects and suggest the possibility of delivering let-7 or its mimic as a type of RNA therapy for T2D. Like in the mouse, the human let-7 family separates into many clusters. So far, no genetic information is available that helps pinpoint the ones that are most relevant to metabolic disorders; yet, circulating let-7a was reported to significantly increase after lifestyle intervention in patients with prediabetes (50). Given that TET3 expression is aberrantly elevated in the livers of patients with T2D (12) and that let-7 overexpression represses the TET3/HNF4α-P2 axis and reduces glucose production in primary hepatocytes from obese humans (Fig. 4 *J–L* and *SI Appendix*, Fig. S4 *C–E*), as well as the success of liver-directed gene therapy with AAV vectors in patients with hemophilia B (51–54), targeting the hepatic let-7/TET3/HNF4α-P2 pathway using liver-specific AAV vectors will be highly clinically relevant.

The significance of our findings extends beyond the realm of glucose metabolism and T2D. First, let-7 is widely accepted as a tumor suppressor and down-regulation of let-7 expression has been noted in various types of malignancies (55). Importantly, metformin was shown to increase let-7 expression in breast and pancreatic cancer cells (56). Given its ability to regulate cellular redox, it is possible that a redox/let-7-dependent mechanism might be involved in metformin’s anticancer activity. Second, aberrantly increased expression of TET1 in vascular endothelial cells has been linked to human atherosclerosis (57, 58). Like its family member TET3, TET1 is also a target of let-7-induced inhibition of expression (58). It remains to be investigated whether activation of the redox/let-7 mechanism might contribute to the observed beneficial effects of metformin in treating cardiovascular disease (8). In summary, we have uncovered a major cellular and molecular mechanism of metformin action that may have a broad impact and that may be important for the development of strategies for prevention and treatment of not only T2D but also other chronic diseases.

## Materials and Methods

### Mouse.

All animal work was conducted in accordance with the guidelines of the Yale University Institutional Animal Care and Use Committee. Male HFD (C57BL/6J-DIO, 380050) and ob/ob mice (B6.Cg-Lepob/J, 000632) were purchased from the Jackson Laboratories and housed at 22 °C to 24 °C with a 12-h light/12-h dark cycle with regular chow (Harlan Teklad no. 2018, 18% calories from fat) or HFD (Research Diets, D12451, 45% calories from fat, for HFD mice) and water provided ad libitum. Before experiments, mice were allowed to acclimate for at least 7 d in the animal facility.

### Metformin and GSSG Treatments of Primary Hepatocytes.

Mouse primary hepatocytes were prepared as previously described (12). Cells were seeded in 12- or 24-well plates (Corning BioCoat Cellware, Collagen Type I, 62405-607) and maintained in a complete culture medium (Williams’ Medium [GIBCO, 12551, contains 11 mM glucose] supplemented with 5% fetal bovine serum, 10 mM Hepes buffer [GIBCO, 15630-080], 2 mM L-glutamine [GIBCO, 25030-081], 1% antibiotic-antimycotic [GIBCO, 15240-062], 4 mg/L insulin [GIBCO, 12585-014], and 1 μM dexamethasone [Sigma, D4902]). Metformin and GSSG treatments were carried out in the complete culture medium. Cryoplateable primary human hepatocytes (MTOXH1002, lot 1914850-01, Caucasian female, 53 y old, BMI 38; MTOXH1000, lot 1912392-02, Caucasian male, 37 y old, BMI 39) were purchased from Sigma-Aldrich. Cells were thawed in thawing media (MCHT50, Lonza) and seeded in hepatocyte plating media (MP100, Lonza) in 12-well plates (Corning BioCoat Cellware, Collagen Type I, 62405-607). Metformin treatment and let-7a transfection were performed the next day and followed the same protocol for the mouse primary hepatocytes.

### Transfection of Primary Hepatocytes.

For let-7a transfection experiments, cells were seeded in 24-well (or 12-well) plates at a density of 2 × 10^5^ (or 4 × 10^5^) cells/well for mouse primary hepatocytes and 2.5 × 10^5^ (or 5 × 10^5^) cells/well for human primary hepatocytes the day before transfection. To prepare the let-7a transfection solution for each 24-well group of cells, 3 pmol of miCon or let-7a were mixed with 15 μL of OPTI-MEM (Gibco, 31985-070) by gentle pipetting. In parallel, 1 μL of Lipofectamine RNAiMAX (Invitrogen, 13778-150) was mixed with 15 μL of OPTI-MEM. Following 5 min of incubation at room temperature, the two contents were mixed by gentle pipetting, and the resulting 30 μL of transfection solution was added to one well of cells containing 1 mL of culture media. Media were changed the next day, followed by RNA, protein, GSH/GSSH, and/or glucose output analyses at the time points indicated in the figure legends.

### In Vitro Glucose Production Assay.

Glucose production assays were performed using an Amplex Red Glucose/Glucose Oxidase Assay Kit (Molecular Probes, Invitrogen, A22189), according to the manufacturer’s instructions. Primary hepatocytes grown in 12-well plates were used. On the day of the assay, the culture medium was replaced with 500 μL of glucose-free and phenol red-free Dulbecco's Modified Eagle Medium (DMEM) (Gibco, A14430-01) supplemented with 2 mM L-glutamine and 15 mM Hepes for 2 h. Then, cells were incubated in 240 μL of glucose production medium (glucose-free and phenol red-free DMEM, supplemented with 20 mM of sodium lactate, 2 mM of sodium pyruvate, 0.5% bovine serum albumin, 2 mM of L-glutamine, and 15 mM of Hepes) for 4 h. Supernatants (200 μL) were used for measurements of glucose concentration, which was normalized to total protein content of cells.

### Measurement of GSH/GSSG Ratio.

GSH/GSSG ratios of cells and tissues were determined using the GSH/GSSG kit (Abcam, ab205811) according to the manufacturer’s protocols. For cell samples, cells were seeded in 12-well plates at a density of 5 × 10^5^ cells/well for mouse/human primary hepatocytes the day before the assay. On the day of the assay, cells were washed twice with cold phosphate-buffered saline (PBS), resuspended in 200 μL of ice-cold PBS/0.5% Nonidet P-40, homogenized quickly by pipetting up and down a few times, and then transferred to ice-cold tubes. For tissue samples, 20 mg of previously snap-frozen liver tissues were washed twice with cold PBS, homogenized 5 s in 400 μL of ice-cold PBS/0.5% Nonidet P-40 using a battery-operated pestle motor mixer (Argos, catalog number A0001), followed by centrifugation for 15 min at 4 °C to remove insoluble materials. Supernatants were transferred to ice-cold tubes and treated with Deproteinizing Sample Preparation Kit with trichloroacetic acid (Abcam, ab204708) to remove enzymes that could interfere with the analysis. For GSH detection, 50 μL of fresh GSH Assay Mixture was added to each GSH standard and sample wells; for total glutathione (GSH + GSSG) detection, 50 μL of fresh total Glutathione Assay Mixture was added to each GSSG standard and sample wells. Incubation was carried out at room temperature for 40 min protected from the light. Fluorescence excitation/emission = 485/535 nm was measured using a fluorescence microplate reader (Molecular Devices, FilterMax F5). Readings were applied to the GSH/GSSG standard curves to obtain the GSH/total glutathione concentrations. GSSG concentration = (total glutathione − GSH)/2.

### Metformin Therapy of HFD Mice.

Metformin was purchased from ENZO Life Sciences International Inc (ALX-270-432-G005), and a stock solution (100 mg/mL) was prepared in Millipore water and stored at −20 °C in aliquots. A working solution was freshly prepared every other day by diluting the stock solution in tap water. For experiments shown in Fig. 2, metformin was administrated to HFD mice starting at the age of 18 wk (HFD initiated at age of 6 wk), and metabolic studies were initiated 3 wk later. For experiments shown in Fig. 3, metformin was administrated to HFD mice starting at the age of 15 wk (HFD initiated at age of 6 wk), and metabolic studies were initiated 3 wk later. PTTs, GTTs, and ITTs were conducted with 1 wk apart between the tests. Blood and tissue samples were collected following a 3-h morning fasting (8:00 AM to 11:00 AM). Tissue samples were harvested following a transcardial perfusion with PBS, snap-frozen in liquid nitrogen, and stored at −80 °C.

### In Vivo Virus Administration.

The AAV8-let-7-TuD virus was custom designed and produced by SignaGen Laboratories. AAV8-let-7a Amm1000108) and AAV8-vec (Am00100) were purchased from Applied Biological Materials Inc. For experiments shown in Fig. 3, HFD mice with or without metformin treatment at the age of 15 wk (HFD initiated at age of 6 wk) were tail vein injected with AAV8-let-7-TuD or AAV8-vec. For experiments shown in Fig. 4, ob/ob mice at the age of 5 wk were tail vein injected with AAV8-let-7a or AAV8-vec. All viruses were injected at 2 × 10^10^ gc/mouse in 150 μL of Dulbecco’s PBS (calcium/magnesium-free, Gibco, 14190144). The tail was cleansed with 70% ethanol, and the injection was made in the lateral vein, using 30-gauge needles.

### Measurement of Metformin Concentrations.

Metformin concentrations from previously frozen plasma and liver tissues isolated from HFD mice were determined using liquid chromatography with tandem mass spectrometry as previously described by us (15).

## Supplementary Material

Supplementary File

## Data Availability

All study data are included in the article and/or *SI Appendix*.
