# Supplementary material for: Let-7 underlies metformin-induced inhibition of hepatic glucose production
Source: Proc Natl Acad Sci U S A. 2022 Mar 28;119(14):e2122217119. doi: 10.1073/pnas.2122217119 (PMC9169108; doi:10.1073/pnas.2122217119)
Supplement: Supplementary File [file pnas.2122217119.sapp.pdf]

Supplementary Information for

Let-7 underlies metformin-induced inhibition of hepatic glucose production.

Di Xie[a,2], Fan Chen[a,2,3], Yuanyuan Zhang[a,2,4], Bei Shi[b], Jiahui Song[c], Kiran Chaudhari[d], Shao-Hua Yang[d], Gary J. Zhang[a], Xiaoli Sun[a,5], Hugh S. Taylor[a], Da Li[c,1], Yingqun Huang[a,1]

[1]Corresponding authors. Yingqun Huang and Da Li

Email: yingqun.huang@yale.edu; leeda@ymail.com

This PDF file includes:

Supplementary Materials and Methods

Figures S1 to S4

Tables S1 to S4

Supplementary Materials and Methods

Western blotting analysis. Primary hepatocytes were homogenized in situ using a pipette tip in 2x SDS-sample buffer with 10% β-mercaptoethanol at room temperature in less than 5 sec followed by heating at 100°C for 5 min with occasional vortexing. Frozen liver tissue samples (~50 mg) were homogenized in 200 μl of tissue lysis buffer (15% SDS, 75 mM Tris-HCl, pH 7.4, 1x Protease inhibitor cocktail [Thermo, 78438], 1x Phosphatase inhibitor cocktail [Thermo, 78427], 5% β-mercaptoethanol) using a BeadBug6 Microtube homogenizer (Benchmark) set at speed 3600, 20s on and 20s off for 6 cycles. The lysate was cooled down on ice for 10 min, followed by centrifugation at 12,000 g at 4 ^{0}C for 7 min to remove insoluble materials. Supernatant was transferred to a new tube and glycerol (final concentration 20%) and Bromophenol (for tracking purpose during gel running) were added. Samples were then heated at 100°C for 5 min with occasional vortexing, aliquoted, and stored at -80 °C until use. For Western blotting, samples were freshly diluted at 1:1 – 1:3 in 2xSDS-sample buffer before loading, and 5-10 µl per well were loaded onto a 4-15% gradient SDS gel (Bio-rad), followed by Western blotting analysis. Image J was used to quantify the protein bands.

RNA extraction and RT-qPCR assays. Total RNA was extracted from primary hepatocytes or liver tissue samples using PureLink RNA Mini Kit (Ambion, 12183018A). For mRNA PCR, 0.8 µg of total RNA was reverse transcribed to cDNA in a reaction volume of 20 µl using PrimeScript RT Reagent Kit (TAKARA, RR037A). Quantitative real-time PCR reactions were carried out using iQSYBRGreen (Bio-Rad) in a Bio-Rad iCycler. Gene expression levels were normalized against housekeeping genes HPRT1 and RPLP0. The specific PCR primers for mouse and human were summarized in *SI Appendix* Table S3. For let-7 PCR, 0.8 µg of total RNA was reverse transcribed to cDNA in a reaction volume of 20 µl using miScript II RT kit (QIAGEN, 218161). Quantitative real-time PCR reactions were carried out using miScript SYBR Green PCR Kit (QIAGEN, 218073) in a Bio-Rad iCycler. Gene expression levels were normalized against RNU6-2-11. The specific PCR primers were summarized in *SI Appendix* Table S4.

Blood chemistry. Blood samples were collected in EDTA tubes (Microtainer with K_2EDTA, BD, 365974) by cardiac puncture of terminally anesthetized animals. The tubes were centrifuged at 2,000 x g at 4 ^{0}C for 20 min, and supernatant plasma was collected and stored at -80 ^{0}C until use. Kits used to measure alanine transaminase (EALT-100), aspartate transaminase (EASTR-100), and lactate dehydrogenase (DLDH-100) were purchased from Bioassay Systems. The bilirubin assay kit (MAK126) was purchased from Sigma Aldrich.

PTT, GTT, and ITT. Pyruvate tolerance tests (PTT) and glucose tolerance tests (GTT) were performed following 16 h overnight fasting. Each animal received an i.p. injection of 2 g/kg pyruvate (Sigma-Aldrich, cat#P5280) or 2 g/kg of glucose (Sigma-Aldrich, G5767) in sterile saline. Insulin tolerance tests (ITT) were performed following a 3 h morning-fasting. Each animal received an i.p. injection of 1 U/kg insulin (Novolin R Regular U-100 insulin). Blood glucose concentrations were measured using Contour next blood glucose meter (Ascensia Diabetes Care) via tail vein bleeding at the indicated time points after injection. For all experiments, age-matched animals were used. For information on animal numbers, refer to figure legends.

Statistical Methods. The number of independent experiments and the statistical analysis for each figure are indicated in the legends. All statistical analyses were performed using GraphPad Prism version 8 for Windows (GraphPad Software, La Jolla California USA, www.graphpad.com) and are presented as mean ± SEM. Two-tailed Student's *t* tests (or as otherwise indicated) were used to compare means between groups. $P<0.05$ was considered significant.

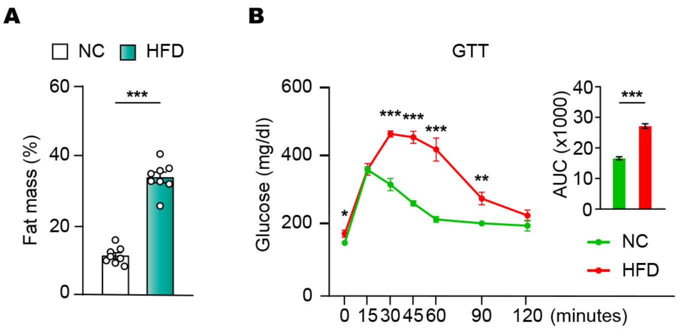

Figure S1. Body fat mass (*A*) and GTT (*B*) of mice fed on normal chow (NC) or HFD at the age of 18-weeks. HFD was initiated at the age of 6 weeks and lasted for 12 weeks. Diabetes were confirmed by increased fasting glucose and decreased glucose tolerance in the HFD group. Data are presented as mean of SEM (n=8 animals in each group). *, P < 0.05; **, P < 0.01; ***, P < 0.001. *A*, Two-tailed Student's *t* tests. *B*, Two-way ANOVA with Sidak post-test. AUC, area under the curve.

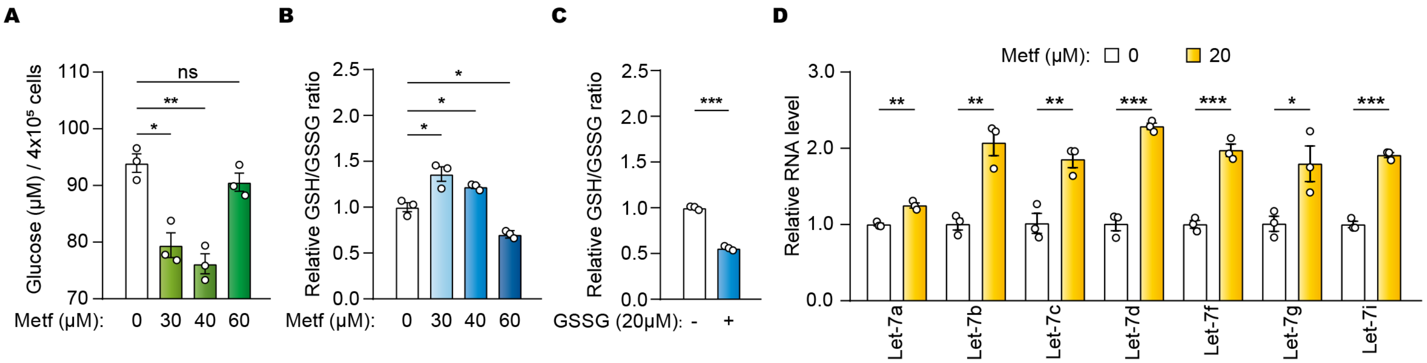

Figure S2. Metformin regulates redox, let-7, and glucose production from hepatocytes isolated from ob/ob mice at the age of 8-weeks. (*A*) Glucose production by ob/ob hepatocytes at 40 h following incubation with metformin at the indicated concentrations. (*B*) Relative GSH/GSSG ratio in ob/ob hepatocytes treated with metformin at the indicated concentrations for 18 h. (*C*) qPCR of let-7 isoforms in ob/ob hepatocytes following treatment with metformin for 24 h. All data are representative of at least two independent experiments. Error bars are mean with SEM of technical replicates (n=3). *, P < 0.05; **, P < 0.01; ***, P < 0.001; ns, nonstatistical significance. (*A* and *B*), One-way ANOVA with Dunnett post-test; (*C*), Two-tailed Student's *t* tests.

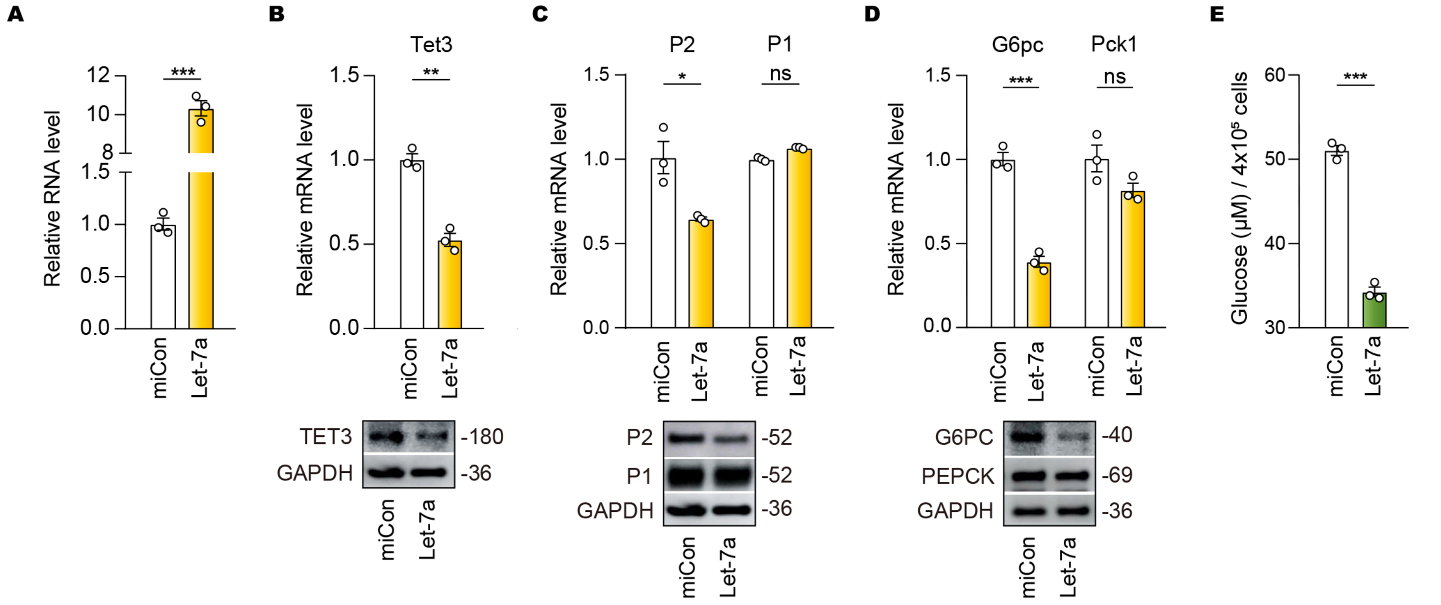

Figure S3. Let-7a overexpression suppresses TET3/HNF4α-P2 and HGP in ob/ob hepatocytes. (*A*) qPCR of let-7a in ob/ob hepatocytes transfected with miCon or let-7a for 24 h. (*B*) qPCR and Western blotting analyses of TET3 in ob/ob hepatocytes transfected with miCon or let-7a for 38 h. (*C*) qPCR and Western blotting analyses of HNF4α-P2 and HNF4α-P1 in ob/ob hepatocytes transfected with miCon or let-7a for 38 h. (*D*) qPCR and Western blotting analyses of G6PC and PEPCK in ob/ob hepatocytes transfected with miCon or let-7a for 38 h. (*E*) Glucose production by ob/ob hepatocytes transfected with miCon or let-7a for 40 h. All data are representative of two independent experiments and are presented as mean ± SEM of technical replicates (n=3). *P < 0.05, **P < 0.01, ***P < 0.001. ns, nonstatistical significance. Two-tailed Student's *t* tests.

Figure S4

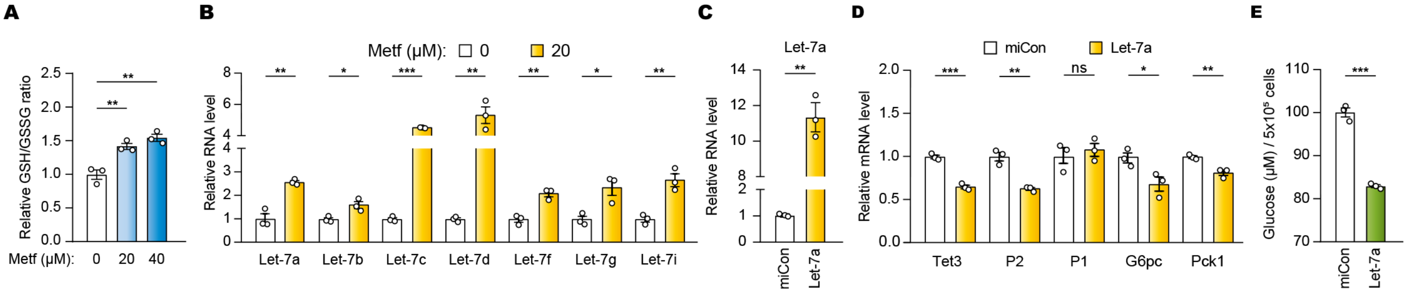

Figure S4. Regulation of glucose production by the let-7/TET3/HNF4a-P2 axis in primary hepatocytes isolated from a male obese human subject. (*A*) Relative GSH/GSSG ratio in primary hepatocytes treated with indicated concentrations of metformin for 18 h. n=3, technical replicates, One-way ANOVA with Dunnett post-test. (*B*) qPCR of let-7 in primary hepatocytes treated with metformin for 24 h. n=3, technical replicates, Two-tailed Student's *t* tests. (*C*) qPCR of let-7a in primary hepatocytes transfected with miCon or let-7a for 24 h. n=3, technical replicates, Two-tailed Student's *t* tests. (*D*) qPCR of indicated genes in primary hepatocytes transfected with miCon or let-7a for 36 h. n=3, technical replicates, Two-tailed Student's *t* tests. (*E*) Glucose production by primary hepatocytes transfected with miCon or let-7a for 38 h. n=3, technical replicates, Two-tailed Student's *t* tests. All data are presented as mean of SEM. *, P < 0.05; **, P < 0.01; ***, P < 0.001; ns, not statistically significant.

Table S1. Metformin concentrations

	Metformin in plasma (μM)	Metformin in liver (μM)
Mouse 1	5.56	16.99
Mouse 2	16.38	42.89
Mouse 3	7.90	31.38
Mouse 4	4.42	13.30
Mouse 5	5.33	21.94
Mouse 6	11.27	76.86
Mouse 7	9.37	40.85
Mouse 8	8.84	41.30
Mean	8.63	35.69
SEM	1.38	7.15

Table S2. Serum biochemistry parameters

	Veh + AAV8-vec	Metf + AAV8-vec	Metf + AAV8-let-7-TuD
ALT (U/L)	40.46 (±4.96)	37.76 (±4.07)	37.80 (±4.27)
AST (U/L)	80.29 (±5.10)	47.71 (±2.61)	53.61 (±6.19)
LDH (IU/L)	75.95 (±3.05)	63.06 (±3.60)	67.56 (±7.53)
Bilirubin (mg/dL)	0.86 (±0.03)	1.12 (±0.05)	1.16 (±0.05)

Plasma enzymatic activities of alanine transaminase (ALT), aspartate transaminase (AST), lactate dehydrogenase (LDH), and bilirubin levels from HFD mice at 4.5 weeks after the indicated treatments. n=8 in each group.

Table S3. Primer sequences

**qPCR primer sequences (mouse)**		
**Gene**	**Forward Primer**	**Reverse Primer**
Rplp0	5'-GATGGGCAACTGTACCTGACTG-3'	5'-CTGGGCTCCTCTTGGAATG-3'
Hprt1	5'-CAGTCCCAGCGTCGTGATTA-3'	5'-GGCCTCCCATCTCCTTCATG-3'
G6pc	5'-ATCCGGGGCATCTACAATG-3'	5'-TGGCAAAGGGTGTAGTGTCA-3'
Pck1	5'-TGTTTACTGGGAAGGCATCG-3'	5'-AGGTCTACGGCCACCAAA-3'
HNF4a-P1	5'-ATGGGCAATGACACGTCC-3'	5'-CTCACGCTCCTCCTGAAGAA-3'
HNF4a-P2	5'-GCGAGTCCTTATGCCCTCA-3'	5'-TGAATTGAGGTTGGCACCTT-3'
Tet3	5'-CTTCCTATGGCTGGGAGTGAG-3'	5'-CTGCCTTGAATCTCCATGGTAC-3'
**qPCR primer sequences (human)**		
**Gene**	**Forward Primer**	**Reverse Primer**
HPRT1	5'-GACCAGTCAACAGGGGACAT-3'	5'-CCTGACCAAGGAAAGCAAAG-3'
RPLP0	5'-GGCGACCTGGAAGTCCAACT-3'	5'-CCATCAGCACCACAGCCTTC-3'
G6PC	5'-CCTCAGGAATGCCTTCTACG-3'	5'-TCTCCAATCACAGCTACCCA-3'

PCK1	5'-GGTTCCCAGGGTGCATGAAA-3'	5'-CACGTAGGGTGAATCCGTCAG-3'
HNF4a-P1	5'-GGTTTGAAAGGAAGGCAGAG-3'	5'-AGGGTGGTGTAGGCTGGGTC-3'
HNF4a -P2	5'-CTTACGGTCTGCAGTTTCCA-3'	5'-ACATCCTCCTCCTGCTGCTA-3'
TET3	5'-GACGAGAACATCGGCGGCGT-3'	5'-GTGGCAGCGGTTGGGCTTCT-3'

Table S4. Key resources table

REAGENT or RESOURCE	SOURCE	IDENTIFIER
Antibodies		
Anti-TET3	GeneTex	GTX121453
Anti-G6PC	Abcam	ab83690
Anti-PEPCK	Abcam	ab70358
Anti-HNF4α-P1	Abcam	ab41898
Anti-HNF4α-P2	Bio-Techne	PP-H6939-00
Anti-GAPDH	Cell Signaling	C67H9
Anti-rabbit IgG, HRP-linked	Cell Signaling	7074
Anti-mouse IgG, HRP-linked	Cell Signaling	7076
Biological Samples		
Primary human hepatocytes	Sigma-Aldrich	MTOXH1002<br>MTOXH1000
Chemicals, Peptides, and Recombinant Proteins		
Phosphatase inhibitor cocktail	Thermo	78427
Protease inhibitor cocktail	Sigma-Aldrich	78438
Dexamethasone	Sigma-Aldrich	D4902
Insulin	Gibco	12585-014
Novolin R human insulin (100 units/ml)	Novo Nordisk	N/A
Williams Medium	Gibco	12551
GSSG	Sigma	G4376-1G
D-(+)-Glucose	Sigma-Aldrich	G5767
Sodium pyruvate	Sigma-Aldrich	P5280
Sodium lactate	Sigma-Aldrich	71718
Critical Commercial Assays		
Amplex Red Glucose Assay Kit	Molecular Probes, Invitrogen	A22189
GSH/GSSG Kit	Abam	ab205811
Bilirubin Assay Kit	Sigma-Aldrich	MAK126
Alanine Transaminase Assay Kit	Bioassay Systems	EALT-100
Aspartate Transaminase Assay Kit	Bioassay Systems	EASTR-100
Lactate dehydrogenase Assay Kit	Bioassay Systems	DLDH-100
Lipofectamine RNAiMAX reagent	Invitrogen	13778-150
PrimeScript RT Reagent Kit	TaKaRa	RR037A
miScript SYBR Green PCR kit	Qiagen	218073
miScript II RT kit	Qiagen	218161
SYBR Green PCR Master Mix	Bio-Rad	172-5124
PureLink RNA Mini Kit	Ambion	12183018A
Experimental Models: Organisms/Strains		
ob/ob mouse: B6.Cg-Lepob/J	The Jackson Laboratory	000632
HFD mouse: C57B/6J-DIO	The Jackson Laboratory	380050
Oligonucleotides and siRNAs		

Primers for qPCR see Supplementary Table 2	This paper	
let-7a (let-7a-5p mimic)	Active Motif	MIM0001
miCon (non-targeting miRNA mimic)	Active Motif	MIM9001
Let-7 isoform and U6 PCR primers:<br>let-7a-2<br>let-7b-1<br>let-7c-1<br>let-7d-1<br>let-7f-1<br>let-7g-2<br>let-7i-1<br>RNU6-2-11	Qiagen	 MS00032179<br>MS00001225<br>MS00005852<br>MS00001232<br>MS00005866<br>MS00010983<br>MS00001253<br>MS00033740
Viruses		
AAV8-let-7a (GFPmmu-let-7a-5p AAV serotype 8)	Applied Biological Materials	Amm1000108
AAV8-vec (empty vector AAV serotype 8)	Applied Biological Materials	Am00100
AAV8-let-7-TuD	This paper	
Software and Algorithms		
ImageJ	Schneider et al., 2012	https://imagej.nih.gov/ij/
Prism 8	Graphpad	https://www.graphpad.com/scientificsoftware/prism/
